# Mediation effects of positive and negative affect on the relationship between emotional intelligence and life satisfaction in rural school teachers

**DOI:** 10.3389/fpsyg.2023.1129692

**Published:** 2023-04-24

**Authors:** Xiaoxiang Deng, Jiasheng Chen, Yuyang Zhao

**Affiliations:** ^1^National Institutes of Educational Policy Research, East China Normal University, Shanghai, China; ^2^School of Social Development, East China Normal University, Shanghai, China; ^3^Department of Social Work, School of Sociology and Political Science, Shanghai University, Shanghai, China

**Keywords:** emotional intelligence, positive affect, negative affect, life satisfaction, mediation effects

## Abstract

Few studies have investigated the ways in which the specific facets of trait emotional intelligence (EI), positive affect (PA), and negative affect (NA) influence individuals’ general life satisfaction, especially in teachers. This study explored the effects of three facets of trait EI [appraisal and expression of emotions (AEE), utilization of emotion (UE), and regulation of emotions (RE)] and two typical affects (PA and NA) on teachers’ general life satisfaction. The participants were 577 Chinese rural school teachers (ages 18–49 years) who completed three questionnaires—the Schutte Self-Report Emotional Intelligence Test, Positive and Negative Affective scale, and Satisfaction with Life Scale. After validating the scales, a structural equation modeling analysis showed that trait EI, PA, and NA had a significant and positive effect on teachers’ general life satisfaction. PA played a partial mediating role between trait EI and life satisfaction. Furthermore, this study found that PA significantly and positively mediated the relationship between AEE, UE, RE, and life satisfaction. These results suggest that teachers with higher EI are more likely to have positive emotions, thereby enhancing their general life satisfaction, and that understanding the role of one’s own and others’ emotions and increasing positive emotions may be the key to improving teachers’ general life satisfaction. Future implications and the study limitations are discussed.

## Introduction

Over the past two decades, the use of emotional skills has been increasingly linked to positive life outcomes in many theoretical and empirical studies (MacCann et al., 2020). Additionally, previous analyses have demonstrated reliable associations between different emotional intelligence (EI) instruments and health indicators (Schutte et al., 2007; Martins et al., 2010), indicating that the skillful use of emotions can lead to higher rates of positive emotional states and reduced negative emotions. In this regard, some studies have demonstrated a link between EI and life satisfaction (LS) and concluded that the former can help people better understand associated psychological processes, such as LS and positive emotions (Zeidner et al., 2012). Related research has suggested that those with higher levels of EI are better able to cope with daily stress and difficulties, form lasting relationships, and experience greater social support (Zeidner et al., 2012). In addition, Schneider et al. (2013) found that those with higher EI are more resilient, allowing them to better adapt to changes under stressful conditions and view stress as an opportunity rather than a threat. Ultimately, having EI enables the use of increased resources and adaptive strategies, leading to long-term emotional benefits (Salovey et al., 2000). Thus, we hypothesize that emotional skills have a two-way effect, i.e., while they can reduce negative emotions in response to stressful events, they can also increase the prevalence and duration of positive emotions over time (Zeidner et al., 2009). Consequently, EI is considered an indicator of psychological adjustment and a major factor in overall well-being (Mayer and Salovey, 1995). Thus, we expect to find significant correlations between EI and LS indicators.

### Emotional intelligence

Salovey and Mayer (1990) first discussed EI by dividing it into the following three categories of adaptive abilities: the appraisal and expression of emotions (AEE), regulation of emotions (RE), and utilization of emotions (UE) in problem-solving. Although emotion is at the heart of this model, it also encompasses social and cognitive functions related to AEE, RE, and UE. Goleman’s (1995) influential book *Emotional Intelligence* introduced many important correlates to EI and extended its structure to some extent to include specific social and communication skills that are influenced by emotional understanding and expression. Cooper and Sawaf’s (1997) well-known book *Executive EQ* outlined an EI model that links specific skills and tendencies through four building blocks: emotional literacy, well-being, depth, and alchemy. Beginning with the origin of EI as a concept, it has been understood as a set of interrelated abilities (Salovey and Mayer, 1990; Mayer and Salovey, 1997; MacCann et al., 2014) and an eclectic combination of many personality traits (Petrides and Furnham, 2003; Boyatzis and Sala, 2004; Tett et al., 2005; Bar-On, 2006), but the term EI came to be used to cover many different characteristics and concepts (Zeidner et al., 2004; Landy, 2005; Murphy and Sideman, 2006), leading to considerable confusion and misunderstanding of what EI is or should be (Ashkanasy and Daus, 2005; Gohm et al., 2005; Mayer, 2006).

To clarify the concepts in this field, researchers have developed two measurement models: ability EI scales and self-rating EI scales (Mayer et al., 2000). Ability EI scales require test-takers to demonstrate their knowledge by providing responses based on emotion-related information, whereas self-rating EI scales require participants to rate their agreement with a series of statements about themselves, thus reflecting their EI level. In related research, self-reported measures of EI were found to be more predictive of social functioning performance than performance measures (Brackett et al., 2006). In addition to the distinction between the two measurement models, there is a similar distinction between two theoretical EI models: mixed models and ability models. Mixed models primarily point to a broad compositional structure that leads to emotionally intelligent behavior, including emotion-related abilities, personality traits, and motivational factors (Bar-On, 2006; Petrides et al., 2007). Conversely, ability models define EI as a cognitive ability similar to language or numeracy but with its content focused on emotions rather than words or numbers (MacCann et al., 2014). Ashkanasy and Daus (2005) further distinguished between two rating scales: ratings of EI abilities and ratings of mixed models. The former are sometimes referred to as “self-perceptions of EI and emotional self-efficacy” (Qualter et al., 2012). Moreover, a recent meta-analysis divided EI measurement into ability, self-rated, and mixed EI (MacCann et al., 2020). In this study, we argue that there are two distinct types of assessments based on theoretical and empirical evidence (Joseph and Newman, 2010; O’Boyle et al., 2011): ability EI and mixed EI. The following describes the main ability models of EI and major mixed models of EI.

Mayer and Salovey (1997) developed a modified EI model consisting of the following four branches of EI: (a) perception, appraisal, and expression of emotions; (b) emotional facilitation of thinking; (c) understanding, analyzing, and employing emotional knowledge; and (d) reflective RE to promote emotional and intellectual development. Based on these four branches, the Mayer–Salovey–Caruso Emotional Intelligence Test (MSCEIT) became the best-known assessment of ability EI (Mayer et al., 2003). Additionally, there are some alternative measurements for only one or two of the four branches (e.g., Freudenthaler and Neubauer, 2007; MacCann and Roberts, 2008) as well as the predecessor of the MSCEIT (i.e., the MEIS; Mayer et al., 2000). The youth versions of the MSCEIT and MEIS have frequently been used in research in the field of schooling (Rivers et al., 2012). The Schutte Self-Report Emotional Intelligence Test (SREIT) developed by Schutte et al. (1998) was one of the earliest scales to assess self-reported EI. Specifically, the SREIT is a 33-item scale based on an earlier definition of EI than the four-branch hierarchical model proposed by Salovey and Mayer (1990). Wong’s Emotional Intelligence Scale is another measure of EI that utilizes this earlier definition with four components: perceiving one’s own emotions; perceiving others’ emotions; using emotions; and managing emotions (Law et al., 2004). Subsequently, the Self-Rating Emotional Intelligence Scale was developed to accurately capture the four-branch model of EI. This 19-item scale consists of 5 subscales: perceiving emotions; using emotions; understanding emotions; managing one’s own emotions; and managing others’ emotions (Brackett et al., 2006).

Appraisal and Expression of Emotions theoretically includes the ability to recognize one’s own emotions (i.e., internal stimuli); the ability to express emotions in response to external stimuli; the ability to accurately express one’s emotions; the ability to distinguish between true and false emotions; and the knowledge of display rules for emotional expression in different cultural contexts (Salovey and Mayer, 1990; Mayer and Salovey, 1997; Mayer et al., 2016). However, in actual tests of EI, AEE is primarily used to measure an individual’s capacity to identify the type and intensity of an emotion present in external stimuli, such as facial expressions, micro expressions, tone-of-voice, body postures, landscapes, and evocative art (Nowicki and Duke, 1994; Matsumoto et al., 2000; Mayer et al., 2003; Schlegel et al., 2014). Once an emotion is perceived, it serves as an input for cognitive systems (Mayer et al., 2001). In this regard, individuals can understand the domain-specific knowledge of emotional content, which includes knowledge about emotions and emotion-related phenomena. According to Mayer et al. (2001), AEE is the central locus for abstract processing and reasoning about emotions and emotional information, including a vocabulary of emotional terms, the antecedents and consequences of emotions, the ways in which emotions may combine or change over time, and the possible effects that particular situations may have on an individual’s present or future emotions (Salovey and Mayer, 1990; Mayer and Salovey, 1997; Mayer et al., 2016).

Regulation of Emotion refers to regulating one’s own and others’ emotions to achieve desired outcomes, such as personal development (Mayer and Salovey, 1997; Mayer et al., 2001, 2016). Because it involves managing one’s own and others’ emotions (i.e., intrinsic and extrinsic RE; Gross and Thompson, 2007; Gross, 2008), this facet includes knowledge about RE and related cognitive strategies (Mayer et al., 2016). RE is also based on personal goals, such as upregulating and downregulating emotions to achieve personal growth. Hence, RE not only represents knowledge regarding how to regulate emotions but also serves as an important motivating factor in deciding when and why to regulate emotions (Mayer et al., 2001).

Utilization of Emotion refers to using emotion and affective information as an input or guidance for cognitive tasks or decision-making. It involves using existing emotions to guide task selection or approaches and generate new emotions to help accomplish specific tasks (Mayer et al., 2016). In this case, positive emotions can lead to innovative thinking because they give individuals a broad perspective, whereas anxiety is a form of hypervigilance to threats, i.e., when using existing emotions, individuals use their current emotional state as a key reference (Fredrickson, 2001; Bar-Haim et al., 2007). Additionally, tasks can be chosen to take advantage of emotional states, which may contribute to performance. For instance, one might choose to welcome new colleagues when in a good mood but wait to attend a funeral or other solemn occasion until the mood becomes more serious.

### Emotional intelligence and life satisfaction

LS, an important aspect of individual subjective well-being (Kjell et al., 2016), reflects an individual’s overall evaluation of their life experience (Diener et al., 2002; Schimmack et al., 2004). Factors influencing LS have been discussed in previous studies, including financial status (Johnson and Krueger, 2006; Boyce et al., 2010; D’Ambrosio et al., 2020), educational level (Kousha and Mohseni, 1997; Meeks and Murrell, 2001; Park et al., 2020), and self-esteem (Utsey et al., 2000; Diener and Diener, 2009; Liang et al., 2020). The factors influencing LS have attracted the attention of researchers because it has a significant impact on physical and psychological health (Strine et al., 2008; Garcia and Moradi, 2013; D’Ambrosio et al., 2020) and is an important component of subjective well-being (Diener, 1984). Positive affect (PA) and subjective well-being have been found to predict greater job dedication and loyalty as well as lower rates of burnout and turnover (Rhoades and Eisenberger, 2002). Rural teachers are generally less satisfied with life than urban teachers (Qiao and Lina, 2019). In view of the work pressures that teachers face, exploring the factors influencing their LS and examining PA and negative affect (NA) will help them better cope with their daily life and work.

EI plays a nonnegligible role among the many factors that affect LS. Individuals with high trait EI report higher LS (Urquijo et al., 2016). This positive association remains significant after controlling for many sociodemographic variables (e.g., Extremera and Fernández-Berrocal, 2005; Gohm et al., 2005; Ruiz-Aranda et al., 2014). Another study found that trait EI is more strongly associated with LS than performance-based EI (Sánchez-Álvarez et al., 2016). The conceptualization and assessment of EI are controversial, but two main ideas are accepted. The first views EI as a set of skills, similar to cognitive intelligence. For instance, Salovey and Mayer (1990) defined ability EI as “the subset of social intelligence that involves the ability to monitor one’s own and others’ feelings and emotions to discriminate among them and to use this information to guide one’s thinking and actions” (p. 189). The second approach primarily considers EI as a set of traits, similar to personality, with Petrides et al. (2007) defining trait EI as “a distinct and compound construct that lies at the lower levels of personality hierarchies” (p. 283).

Previous research has examined the association between trait EI and LS in diverse population samples, namely, in university graduates (Amdurer et al., 2014), university employees (Hafiz and Chouhan, 2015), and employees of secondary schools. This result was also obtained in groups of teachers (Mehta and Mehta, 2015) and undergraduates (Afolabi and Balogun, 2017). However, little attention has been paid to rural teachers. One study found that the LS of rural teachers in China is generally not high (Qiao and Lina, 2019). Among rural teachers who lack resources and face greater pressure, studying the relationship between LS and trait EI will not only enable the adoption of steps to improve the physical and mental health of rural teachers but also help develop measures to stimulate their enthusiasm for education and teaching.

The positive effects of trait EI on LS are well established, but the exact mechanism of this effect remains unclear. Several studies have identified the mediating role of numerous variables, such as self-evaluation characteristics, perceived stress, and social support (e.g., Zeidner et al., 2012; Kong and Zhao, 2013; Sun et al., 2014; Urquijo et al., 2016). Here, we attempt to identify the mechanisms behind this association from the perspective of PA and NA. In particular, for rural teachers, we investigate how they use their emotional resources and abilities to improve their own LS and the mechanisms behind the improvement (Hypothesis 1).

### Moderators of the emotional intelligence/life satisfaction relationship

#### Positive and negative affect

PA and NA are generally considered related to LS and subjective well-being (Diener et al., 1990, 1991), and the relationship between these two affects and LS has different intensities and effects (Kuppens et al., 2008). It is generally accepted that trait EI is negatively associated with NA and positively associated with PA (e.g., Mikolajczak et al., 2007; Gallagher and Vella-Brodrick, 2008; Mikolajczak et al., 2008; Schutte and Malouff, 2011; Koydemir et al., 2013). What is the relationship between trait EI and affective experience? As a facet of trait EI, RE is considered to be closely related to changes in PA and NA. The reappraisal of PA is related to increased PA, and the suppression of PA is associated with decreased PA and increased NA (Nezlek and Kuppens, 2008). RE strategies of suppression and rumination are associated with increases in NA and decreases in PA, and rumination, reappraisal, distraction, and social sharing are associated with increases in PA (Brans et al., 2013). Furthermore, PA and NA are considered to mediate the relationship between RE strategies and sleep quality (Latif et al., 2019). Diener’s (1984) tripartite expression of subjective well-being is very simple, incorporating LS, PA, and NA. Later studies supported this compositional structure and showed invariance across groups from middle childhood to adulthood (Huebner and Dew, 1996). However, although follow-up studies have confirmed the generality of the association between PA, NA, and LS, the structure linking LS, PA, and NA remains ambiguous (Busseri, 2018). Research conducted among teachers has shown a strong link between PA and LS (Hamama et al., 2013).

Scholars have noticed a strong link between PA and NA as well as LS and EI. PA and NA are envisioned in a model as mediating the link between EI and LS, i.e., people with good EI experience more PA and less NA, leading to higher LS (Zeidner et al., 2012). EI may promote the use of abundant resources and adaptive responses, resulting in long-term emotional benefits (Sánchez-Álvarez et al., 2016). Generally, individuals who experience happiness more often and experience less NA are empirically found to be more satisfied with their lives (Garcia and Moradi, 2013; Koydemir et al., 2013). One study found that EI affects LS through the chain mediation effects of social support-PA and social support-NA and showed that high EI can promote well-being in terms of social support and emotion (Kong et al., 2019). Gignac (2006) used structural equation modeling (SEM) to show that a specific indirect effect of trait EI on LS was significant through PA, but no significant indirect effect was found through NA. Using the same technique, Kafetsios and Zampetakis (2008) showed that PA and NA at work mediate the relationship between EI and job satisfaction. These results suggest that individuals with higher EI scores tend to experience less NA and more PA, leading to increased job satisfaction.

However, some methodological issues have arisen in previous studies. First, Kafetsios and Zampetakis (2008) only tested aggregate indirect effects through PA and NA, not specific indirect effects. Preacher and Hayes (2008) found that the significance of total indirect effects was not a necessary prerequisite for significant specific indirect effects in multiple mediation models. Second, Gignac’s (2006) results were obtained in a very small sample (*N* = 107), which may lead to nonsignificant mediation effects for negative effects. Barrett (2007) asserted that SEM analyses based on a sample of less than 200 should be rejected unless the population itself is small or limited in size. Further, the studies cited above generally discuss trait EI as a single variable, without further exploring how the subdimensions of trait EI and LS are related in terms of the mediating effects of PA and NA. Finally, teachers have been identified as an occupational group that experiences high levels of stress (Greenglass et al., 1997; Van der Doef and Maes, 2002; Pascual et al., 2003), and PA and subjective well-being have been found to predict stronger work dedication and loyalty in addition to lower burnout levels and turnover rates (Rhoades and Eisenberger, 2002). However, the LS of rural teachers is generally not high (Qiao and Lina, 2019). With respect to the work pressure faced by teachers, exploring the impact paths of their LS and positive and negative emotions can help them better cope with daily life and work (Hypothesis 2). 

#### Facets of emotional intelligence

Three potential mechanisms can explain the relationship between EI and LS. First, teachers with high EI may be better equipped to manage negative emotions triggered by the school environment. Common emotions among teachers include burnout, disappointment with negative feedback, learning new technology/topics, and stress from challenging projects (Roeser et al., 2013; Carroll et al., 2022). Teachers with better emotion management skills may also experience LS and professional growth from difficult experiences. If this mechanism is at play, then the emotion management branch of EI should show the strongest correlation with LS.

The second mechanism proposed for the relationship between EI and LS involves social needs in education. Specifically, teachers who possess higher EI may be better equipped to manage their lives and develop better relationships with their students, colleagues, and respective families (Jennings and Greenberg, 2009), which can directly affect LS, such as easier access to resources or improved achievement. Additionally, it can have indirect effects due to the social support network it provides to help manage stress. If this is the case, the RE branch of EI should show stronger effects than the other branches.

Third, MacCann et al. (2014) suggested that EI may be an element of intelligence similar to teachers’ adeptness at languages or other academic subjects, allowing them to become emotionally intelligent. This overlap between EI and teaching skills may explain why teachers with higher EI have higher LS. It is believed then that the larger vocabulary and greater ability to express feelings associated with high EI can help rural teachers manage their daily tasks more effectively and serve as a foundation for their long-term development and career growth.

However, the findings of previous studies have been inconsistent in terms of the relationship between different facets of trait EI and LS. The level of emotional self-efficacy of trait EI (Petrides and Furnham, 2003) may have been the main factor determining the positive direction of its relationship to LS. Emotional self-efficacy directly affects individuals’ emotional state in negative situations and indirectly affects how individuals respond to setbacks and obstacles. However, Palmer et al. (2002) found that regarding trait EI, only clarity of feelings was significantly associated with LS, whereas Liu et al. (2013) found that in an undergraduate student sample, there was no significant correlation between the emotional appraisal of others and LS. A recent study found that one trait EI component (RE) and two adversity quotient facets (tenacity and optimism) significantly and positively predicted general LS (Zhao et al., 2021). Holinka (2015) showed a negative correlation between the interpersonal trait EQ-i (Emotional Quotient-Inventory) subscale and overall LS.

The relationship between trait EI and LS requires further in-depth research. This study explores the relationship between the subdimensions of trait EI and LS, and the results may help researchers in related fields develop a better understanding of facets of trait EI and their relationship to LS (Hypothesis 3). 

## Rural school teachers

In rural areas of central China, teachers have a unique set of circumstances. First, preschool education has been rapidly growing (although not universally), and the government has been working to promptly popularize such education. This indicates that kindergartens and primary schools often include crossover teaching staff, who may transfer from primary school to junior high school or from junior high school to senior high school. This phenomenon reflects the urbanization of education in China and the lack of professional identity among teachers. Second, rural teachers are often responsible for taking care of a large number of “left-behind” children (i.e., children whose parents are not present), which requires significant energy and can create extra strain on the teachers. Furthermore, because many students at these schools are boarders, the class teachers must take on the role of life teachers due to the lack of such dedicated individuals. Finally, due to the shortage of specialists in various subjects, such as music, physical education, and art, rural teachers are often required to provide interdisciplinary lessons. To this end, they must develop a diverse set of new knowledge and skills to support their teaching in addition to their pre-service training, which can be daunting and stressful (Qiao and Lina, 2019). Due to the increased teaching pressure and self-learning challenges faced by rural teachers, it is hypothesized that teachers with higher EI will be better able to cope with such challenges. Furthermore, high EI can help rural teachers increase their positive emotions and decrease their negative emotions. Consequently, they improve their LS, thereby enabling them to stay more active and productive in their daily teaching and fostering long-term benefits for their career development.

## Methods

This study validated the model for the mechanism by which the trait EI of teachers influences their LS through PA and NA and further explored the relationship between the facets of trait EI, PA, and NA in relation to LS. As noted, our study mainly addressed two issues. First, to test the validity of the model, we used a mediation model implemented with SEM to test the simultaneous mediation of PA and NA. Second, because mediation models show the relative importance of mediators (Bravo et al., 2018; Park et al., 2020), the independent mediating roles of PA and NA in the link between trait EI and LS were considered in our study, and we investigated which mediators show a more critical role in the link.

### Participants

A total of 577 participants were included in this study, all of whom were living in county areas in central China, that is, rural areas that are governed under the category of county-type administrative regions in China. Among these, 148 were male (26%) and 429 were female (74%). The average age of the participants was 28.40 years (SD = 6.75). The participants had an average of 15 years of education (SD = 0.627), and most had a university degree (97.57%). Nearly two-thirds were married (65.34%). More than three-quarters of the participants (79%) had no part-time administrative job. The sample was made up of 72.27% teachers at the primary level and 24.96% at the junior high school level. Nearly 60% of the participants were second-level teachers or below, which is representative of the average level of teachers of basic education in China. Thus, the data analysis of these 577 teachers can be regarded as reflecting the psychological characteristics of Chinese rural teachers of basic education. Although the study participants have certain structural differences, there still existed strong homogeneity due to homogeneity among the rural areas of central China. The survey data analysis also reveals some commonalities. In the subsequent analysis, structural differences in terms of subjects such as age, gender, and teaching level were controlled (Table 1).

**Table 1:** Basic statistics of participants.

Outcome	Mean	Standard deviation	Minimum	Maximum
Gender (male = 1)	0.26	0.43	0	1
Marital status (married = 1)	0.65	0.47	0	1
Age	28.40	6.75	18	49
Part-time job or not (no part-time job = 1)	0.79	0.41	0	1
Teaching level (nursery = 1, primary = 2, junior high = 3, senior high = 4)	2.26	0.50	1	4
Years of education	15.05	0.626	9	16

### Data collection

All data used in this study were collected online *via* questionnaires. Three measures were used, one for trait EI, PANAS, and general LS. Additionally, basic demographic information was collected from all participants.

### Measures

In this section, we first evaluate the theoretical context of each core measurement (EI\PA\NA\LS) and then test the reliability and validity of their scales using Bartlett’s test, the Kaiser–Meyer–Olkin (KMO) test, and Cronbach’s alpha coefficient.

#### Emotional intelligence

Trait EI was measured using the SREIT developed by Schutte et al. (1998). SREIT is a short (33-item) self-reported measure of EI developed based on Salovey and Mayer’s (1990) earlier EI model. Schutte et al. (1998) argued that the original EI model of Salovey and Mayer (1990) and the revised EI model of Mayer and Salovey (1997) are the most cohesive and comprehensive models of EI. The former is more suitable for conceptualizing aspects of an individual’s current state of emotional development. The 33-item SREIT, refined by Schutte et al. (1998) on the basis of these models, is succinct, operable, and rigorous. It is precisely for these reasons that we chose trait EI as a tool for testing the relationship between EI and LS.

The overall reliability coefficient of the 33 measurement indicators in this study was 0.938. Additionally, Bartlett’s test value for chi2 was 8,495 (*p* = 0.000), and the KMO test value was 0.947, thus indicating a significant correlation between the indicators. The commonality between the indicators was high, meaning that they could accurately reflect the latent variable EI. In this set of 33 items, the representations of the different categories of the model were roughly proportionate to those in the conceptual model of Salovey and Mayer (1990). In all, 13 items were drawn from among those generated for the AEE category of the SREIT. 10 from among those generated for the RE category, and 10 from among those generated for the UE category. The reliability coefficient of AEE used in this study was 0.807, the chi2 of the Bartlett test was 1738.352 (*p* = 0.000), and the KMO test value was 0.857, indicating that the AEE category had an acceptable degree of homogeneity and commonality. The reliability coefficient for RE was 0.838, the chi2 of the Bartlett test was 1782.662 (*p* = 0.000), and the KMO test value was 0.889, indicating that the internal consistency of the RE category was strong and there was high correlation between items. The reliability coefficient of the UE category was 0.8239, the chi2 of Bartlett test was 1582.466 (*p* = 0.000), and the KMO test value was 0.880, indicating that the UE category had strong internal consistency and the correlation between items was high. Because the scales used in this study were developed and validated in a Western setting, we performed a confirmatory factor analysis (CFA) to ensure that culturally similar constructs were used across all scales, except the CD-RISC, which has been validated against a Chinese population (Yu and Zhang, 2007).

#### Positive and negative affect

The Positive Affect and Negative Affect Scale (PANAS) was compiled by Watson et al. (1988) and revised by Lin et al. (2008). Answers were given on a 5-point scale (1 = very slight or none at all, 5 = very strong) to indicate the extent to which the emotion described was experienced in the previous week, and negative and positive impact scores were calculated separately, with higher scores indicating greater impact. In this study, Cronbach’s alpha coefficients of the PA and NA subscales were 0.949 and 0.949, respectively. Moreover, Bartlett’s test values for chi2 were 5905.597 (*p* = 0.000) and 5488.154 (*p* = 0.000), respectively, whereas the KMO test values were 0.949 and 0.936, respectively. Correlation between the specific items was significant, and the degree of commonality was high, indicating that this instrument can reliably reflect PA and NA intensity of the respondents.

#### Life satisfaction

LS was measured using the Life Satisfaction Scale (SWLS) developed by Diener et al. (1985). The SWLS is a short, 5-item general LS scale wherein participants indicate the extent to which they agree with certain statements using a 7-point Likert scale (1 = strongly disagree to 7 = strongly agree). This study used a 6-point scale; the midpoint of the 7-point scale was removed to avoid midpoint effects or social desirability bias (Garland, 1991). The overall reliability of the five items was good, with an alpha reliability coefficient of 0.949. The alpha of each specific item was greater than 0.9. Moreover, Bartlett’s test value for chi2 was 5488.154 (p = 0.000), whereas the KMO test value was 0.936. Correlation between the specific items was significant, and the degree of commonality was high, which reliably reflects respondents’ overall LS intensity (Table 2).

**Table 2:** Reliability and validity test of core measurements.

	Cronbach’s alpha	KMO test value	Bartlett’s test value for chi2
EI (overall)	0.938	0.947	8495.032^***^
AEE	0.807	0.857	1738.352^***^
RE	0.838	0.889	1782.662^***^
UE	0.824	0.880	1582.466^***^
PA	0.949	0.949	5905.597^***^
NA	0.949	0.936	5488.154^***^
LS	0.949	0.936	5488.154^***^

^***^p < 0.001, ^**^p < 0.01, ^*^p < 0.05.

#### Basic demographic information

The present study collected basic demographic information, including gender, urban–rural category, education level, marital relationship status, and school administrative part-time status.

### Procedure

The Chinese version of the questionnaire used in the study was developed *via* back-translation (Brislin, 1986). First, the original English version was translated into Chinese by two individuals who were fluent in English. Second, the Chinese version was translated back into English by two other individuals who were unfamiliar with the original English version. Third, the translated versions were compared, and adjustments were made to ensure that the Chinese version was comparable with the original English version. Fourth, a panel of five researchers reviewed the Chinese and original English versions to finalize the version used in this study.

The participants were recruited *via* social media in the early fall semester of 2022, during the COVID-19 pandemic. Because the lockdown in China was relatively eased during this period, the teaching life in the school was somewhat normal. Those who were willing to participate in the study were provided with an informed consent form and web link to respond to the questionnaires online. The participants were required to sign the informed consent form before completing the surveys. Participation was voluntary, and participants could withdraw from the process at any time. Because the participants were anonymous, no personal information was collected. No financial compensation was provided to the participants. The study was reviewed and approved by the East China Normal University institutional review board.

### Data analysis

This study adopted a combination of data-driven and theory-driven methods for analyses. First, this study used a data-driven GLASSO model to establish an association network of all variables to present the original relationships among variables. This method has now attracted attention in the field of psychometrics, especially to screen core variables, verify the measurement structure, and explore the relationship among variables (Epskamp et al., 2018; Epskamp and Fried, 2018). In our analysis, we used R Studio to call the GLASSO degree package to implement this function and used the social network analysis tool Gephi to visualize the results of the model. Additionally, to prevent false correlations and multicollinearity among the indicators, the penalty term lambda was set to 0.1 in the model parameter setting.

Second, according to the theoretical framework of the current study and the analysis results of the GLASSO model, we used an SEM to test its core content and ensure the robustness of the core mechanism. In the software tool, we used the Structural Equation Module in STATA 17 to complete the second step analysis.

## Results

### Variable network based on GLASSO model

**Figure 1:**
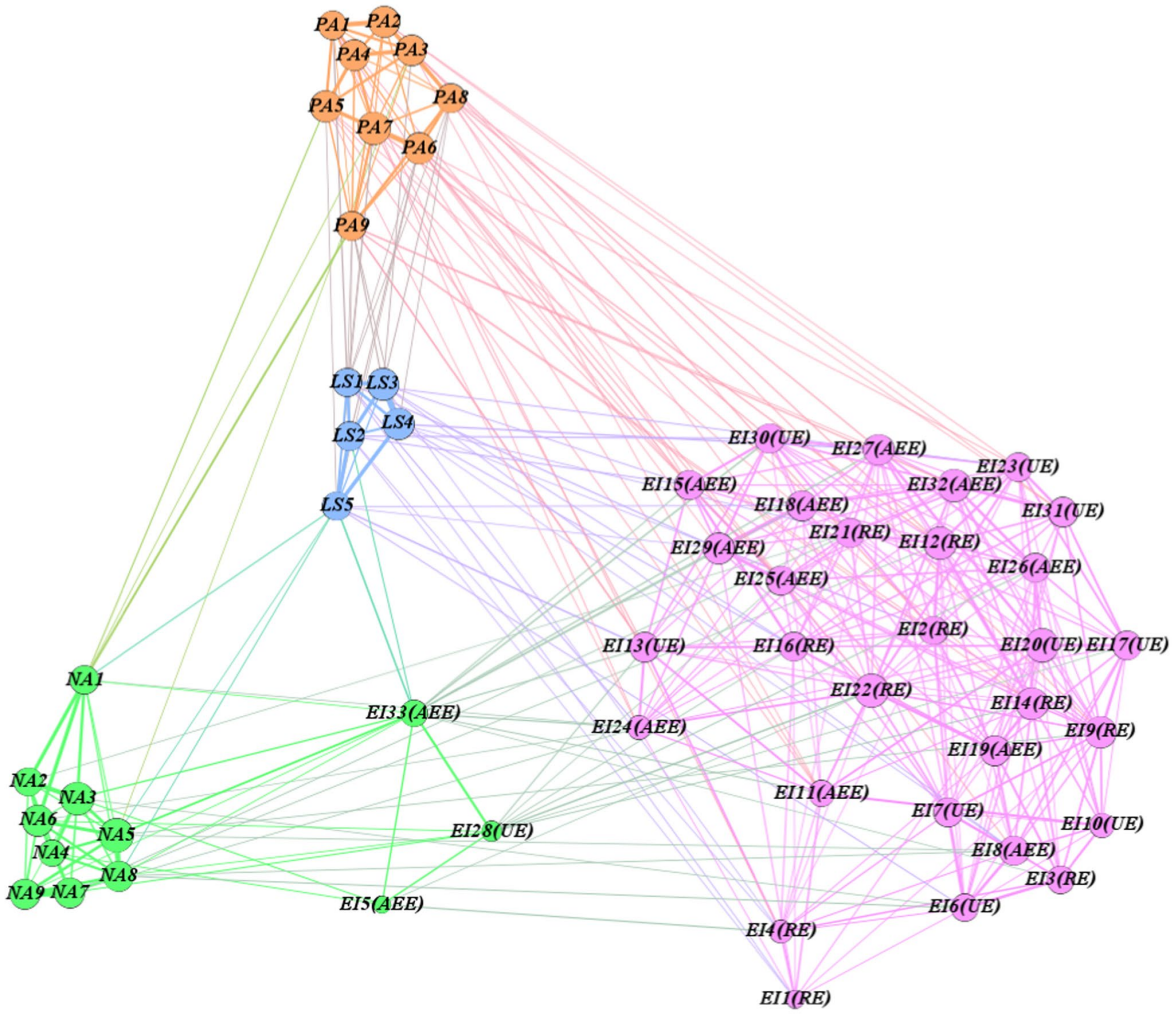
Variable network based on GLASSO model (Lambda = 0.1).

Figure 1 presents the analysis results of the GLASSO model. This model is also a visual representation of the correlation coefficient matrix and the measurements from the reliability and validity tests. Furthermore, it shows the potential structure of each measurement dimension. The network has four subgroups (communities), which correspond to the four types of measurement that this study focused on, and the structure of the four measurements was relatively clear. We found that the EI subgroup influenced the variables of the PA subgroup, which indirectly affected LS. Some EI variables also directly affected LS. However, the network connections between the EI and NA subgroups were relatively sparse, which indicates that the correlation between the two types of variables (EI and NA) was relatively low. Furthermore, there were relatively few connections between NA and LS variables, and the relationship between the two may not be obvious.

In addition, we found that the five variables—EI33, EI28, EI5, EI4, and EI1—had a marginal position in the network, with deviations in their subgroup classification (EI33, EI28, and EI5), thereby indicating that the structural directivity of these five indicators was relatively weak. We tested the five variables using CFA and found that their contributions to the measured latent variables were relatively low, with factor loadings less than 0.4. Therefore, to ensure the accuracy of the following analysis, we eliminate them from the SEM analysis.

### The influence mechanism of EI on teachers’ LS

#### Full model

**Figure 2:**
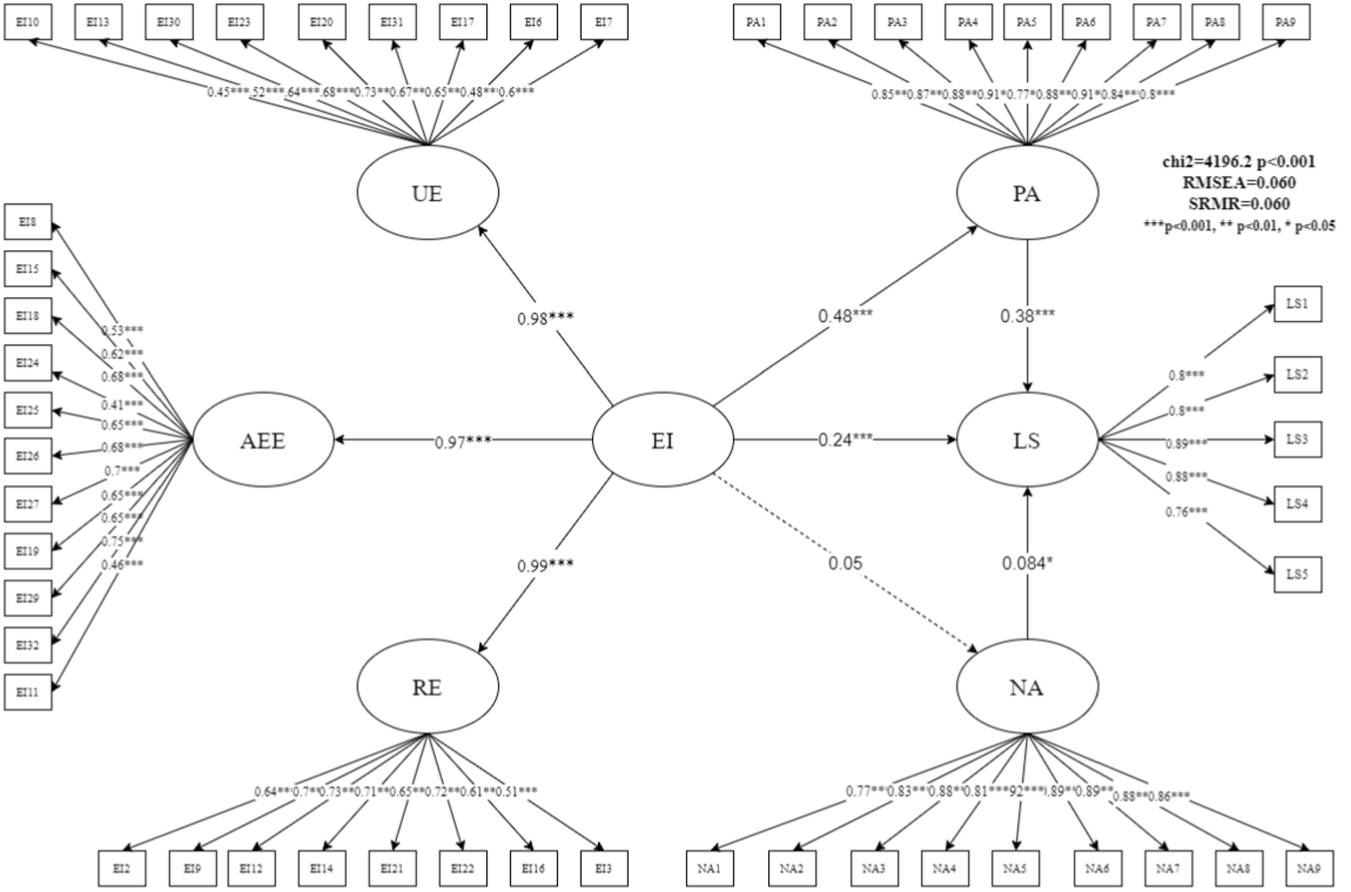
Estimation results of a higher-order factor model of the influence mechanism of teachers’ emotional intelligence.

We used a higher-order factor model in the SEM to verify the complete impact mechanism. Figure 2 shows the estimation results of the full model. The overall fitting of the complete model was acceptable and had a certain explanatory power (RMSEA = 0.06, SRMR = 0.06). Specifically, we found that teachers’ EI significantly affected their PA; its standardized path coefficient was 0.48 (z-value = 14.24, *p* = 0.000), hence indicating that teachers with higher EI were more likely to express PA. The expression of PA also improved teachers’ LS, with a standardized coefficient of 0.38 (z = 8.85, *p* = 0.000). Additionally, EI directly affected teachers’ LS. The standardized coefficient of this path was 0.24 (z = 5.49, *p* = 0.000), that is, teachers with higher EI had higher LS. Therefore, PA partly explained why teachers with different EI had differences in LS; it was an important mediating factor between EI and teachers’ LS, and its mediating effect accounted for 43.18% of the total effect. The intermediary ratio is relatively high. Meanwhile, the effects of EI on NA (z = 1.15, *p* = 0.252) and NA on LS (z = 2.15, *p* = 0.031) were relatively low. Although the influence path of NA as a mediator variable has not been confirmed, this study found that NA had a weak impact on LS in the complete model and its standardized path coefficient was only 0.084. This result is consistent with the results of the GLASSO model, that is, EI influenced teachers’ PA and changed their attitudes toward life.

#### The analysis results of the three subdimensions

**Figure 3:**
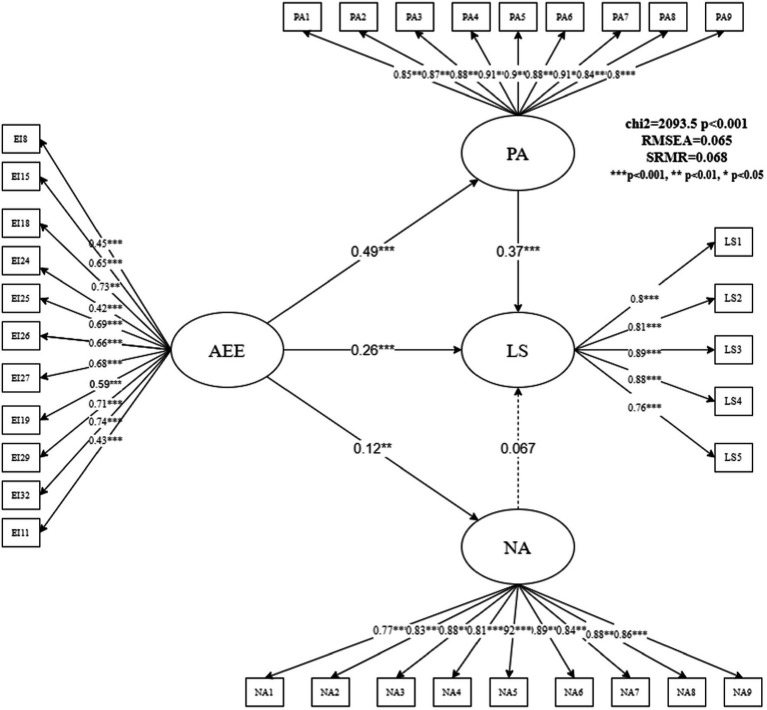
Structural equation model estimation results of AEE affecting life satisfaction.

Figure 3 shows the analysis results of the AEE subdimension model. The fitting index of the model was acceptable (RMSEA = 0.065, SRMR = 0.068). The model results showed that the total effect of AEE on outcome variable LS was 0.44 [0.26 + (0.49 × 0.37)]. PA played an intermediary role between AEE and LS. Improvements to AEE can promote PA, and its normalized path coefficient was 0.49 (z = 13.81, *p* = 0.000). Furthermore, improvement in PA can lead to higher LS among teachers, with a standardized path coefficient of 0.37 (z = 8.70, *p* = 0.000). Therefore, by promoting PA, AEE also promoted teachers’ LS. PA, with a mediating ratio of 41.08%, was an important mediating variable for the AEE and LS path. In addition, the mediating role of NA in this model was not confirmed, and the effect of NA on LS was not significant, whereas the data for the normalized path coefficient of AEE on NA showed a very small value of 0.12.

**Figure 4:**
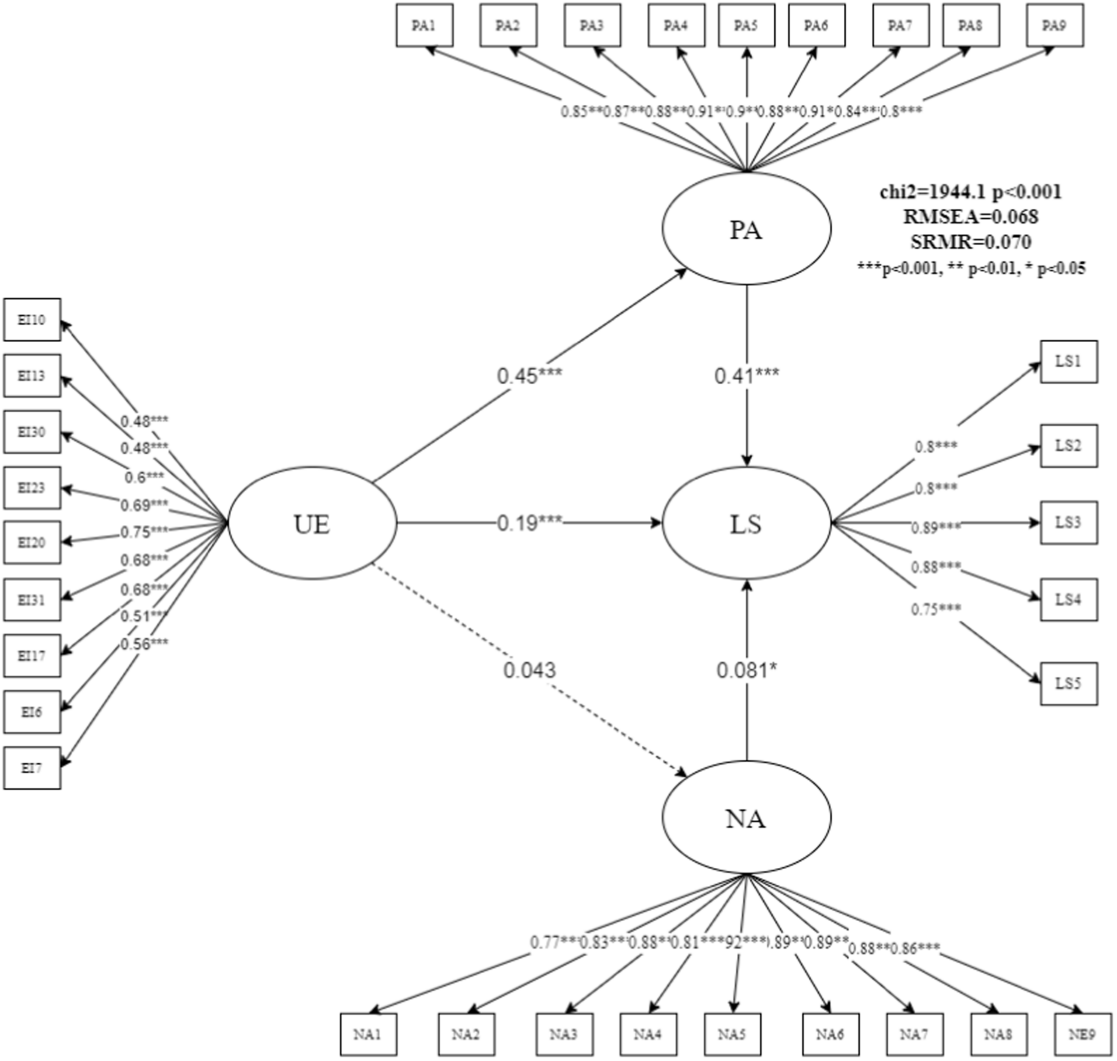
Structural equation model estimation results of UE affecting life satisfaction.

Figure 4 shows the analysis results of the UE subdimension model. The fitting index for the UE model was slightly weaker than that of the AEE subdimension model, with RMSEA = 0.068 and SRMR = 0.07, which are relatively general in terms of the specific index values but may still have certain explanatory significance. The results of the model in Figure 4 show that the total effect of UE on LS was 0.37 [0.19 + (0.45 × 0.41)]. PA was also an important mediating variable in the influence path from UE to LS, and the mediating ratio was 49.27%. Improvement in UE can positively promote PA, with a normalized coefficient of 0.45 (z = 11.78, *p* = 0.000). Furthermore, improvement in PA promoted rural school teachers’ LS, and its standardized coefficient was 0.41 (z = 9.47, *p* = 0.000). Similar to the results of the previous model, the mediating effect of NA was not significant.

**Figure 5:**
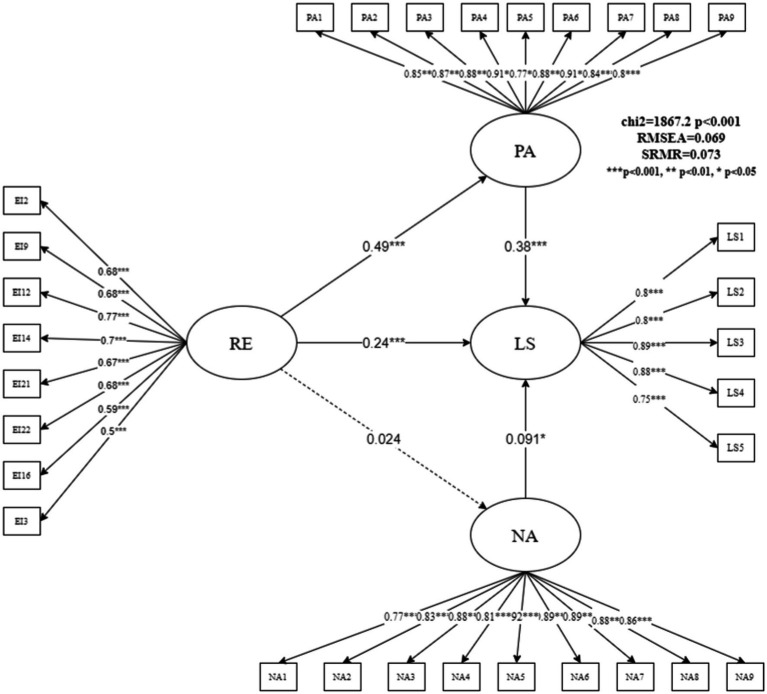
Structural equation model estimation results of RE affecting life satisfaction.

Figure 5 shows the results of the RE subdimension model. The fitting index of the RE model was similar to that of the UE model (RMSEA = 0.069 and SRMR = 0.073). These are relatively general as index values but are important for the topics discussed in this paper. The results of the model in Figure 5 show that the total effect of RE on outcome variable LS was 0.42 [0.19 + (0.45 × 0.41)]. RE indirectly promoted rural school teachers’ LS scores by promoting PA. The normalized path coefficient for RE to PA was 0.49 (z = 13.76, *p* = 0.000), and the path coefficient of PA to LS was 0.38 (z = 8.56, *p* = 0.000). PA played a significant mediating role in this path relationship, with a mediating ratio of 43.69%. Consistent with the results of the previous four models, the mediating effects of NA were not confirmed. NA had a weak effect on LS, and the effect of RE on NA was not significant.

Overall, among the three subdimensions, AEE had the largest overall effect on outcome variable LS, followed by RE, and UE was the smallest. Among these three influence paths, PA played a mediating role in all three subdimension models. The mediating effect of PA was the highest in the UE model, followed by the mediating effect in the RE model, and the smallest in the AEE model. In the three subdimensions, AEE directly affected LS to improve LS. Furthermore, improvement in AEE, RE, and UE promoted PA, which improved teachers’ LS. However, the mediating effect of NA was not confirmed by the data in the full model or the three subdimension models. Therefore, trait EI was more likely to have an indirect impact on teachers’ LS through the regulation of PA or have a direct impact on LS.

*Hypothesis 1*: Overall, higher trait EI is associated with higher levels of LS.

This study examined the differential effects of traits EI, PA, and NA on individual rural teachers’ overall LS. Specifically, we found that self-reported trait EI significantly affected the performance of PA, indicating that higher EI was more likely to be associated with PA and correspondingly, the display of PA also increased LS. Meanwhile, this study found that self-reported trait EI could significantly affect the level of PA and thus influence LS levels, indicating that rural teachers with higher EI may demonstrate higher levels of PA and thus higher LS. Taken together, these findings may contribute to a deeper understanding of the links between these factors by suggesting that trait EI may influence LS primarily from a PA perspective. Therefore, the association between trait EI and LS was confirmed in a large sample of rural school teachers in China, a result that is consistent with the findings in previous literature (e.g., Schutte and Malouff, 2011; Kong et al., 2012; Urquijo et al., 2016; Zhao et al., 2021). These results suggest that EI is an important personal resource for LS.

*Hypothesis 2*: PA and NA play a mediating role between trait EI and LS.

Regarding the second hypothesis, a specific indirect effect of trait EI on LS was demonstrated through PA, supporting the affective mediation model (Zeidner et al., 2012). Furthermore, this finding is consistent with the fact that affective experience is particularly important to judge a person’s LS (Eid and Larsen, 2008; Kuppens et al., 2008; Garcia and Moradi, 2013; Koydemir et al., 2013). Thus, these results are consistent with those of previous studies that highlighted the important role of PA and NA in the EI–LS relationship (Kong and Zhao, 2013; Liu et al., 2013; Kong et al., 2019). Using a mediation model, the results extend previous research by revealing that affective experiences may independently contribute to the EI–LS link in Chinese rural school teacher populations.

We found that PA was the strongest mediator in the relationship between trait EI and LS, thereby suggesting that PA may play a more important role than NA in this relationship. Conversely, the path of trait EI affecting LS through NA was not been supported by the data, which showed that the Chinese rural teacher population in the sample did not change their NA intensity because of their level of EI, which is consistent with Gignac (2006). This finding is broadly consistent with previous results that in addition to social support, resilience quotient, resilience, and so on, PA plays a crucial role in the relationship between trait EI and LS (Kong and Zhao, 2013; Zhao et al., 2021). This may be because people with high EI are better able to perceive, use, and regulate their emotions than those with low EI, thereby experiencing PA with a higher frequency and degree and having a positive emotional life (Sánchez-Álvarez et al., 2016).

*Hypothesis 3*: The three facets of trait EI influence LS through the mediating effects of PA and NA.

Regarding the third hypothesis, most previous studies have considered trait EI to be a single construct associated with LS (e.g., Hafiz and Chouhan, 2015; Mehta and Mehta, 2015; Afolabi and Balogun, 2017) or only found its RE category to be positively correlated with general LS (Zhao et al., 2021). This study found that among the three subdimension models, RE has the best model fit (AIC = 36407.088, BIC = 36707.779). Among the three influence paths, PA played a mediating role in all three subdimension models. Thus, improvements in the three facets of trait EI, namely, the AEE, RE, and UE categories, promoted PA, thereby improving rural school teachers’ LS. The effects of the three facets on NA were small and mostly statistically unconfirmed. This finding validates the proposition that people regulate their own and others’ emotions to achieve personal goals and that achieving these goals will enhance their PA, which may ultimately lead to higher LS (Brackett et al., 2010; Table 3).

**Table 3:** Fitting index of the structural equation model.

	Chi2	RMSEA	SRMR	AIC	BIC
EI (full model)	4196.2^***^	0.060	0.060	60319.451	60546.750
AEE model	2093.5^***^	0.065	0.068	40826.774	40162.328
UE model	1944.1^***^	0.068	0.070	37229.582	37552.062
RE model	1867.2^***^	0.069	0.073	36407.088	36707.779

(1) ^***^p < 0.001, ^**^p < 0.01, ^*^p < 0.05; (2) Because the sample size in this study was 577, which is relatively large, the chi-square test in the fitting index was significant.

## Discussion

### Why does emotional intelligence predict life satisfaction? Insights based on the mediation effects of positive and negative affect

In the Introduction, we suggested three mechanisms that explained why EI could be correlated with LS. First, those with higher EI may be better able to regulate negative feelings, such as anxiety, stress, and disappointment, which are all connected to LS (Roeser et al., 2013; Carroll et al., 2022). If this is true, RE could be responsible for these effects. Second, those with higher EI might be better at navigating their social world and building stronger relationships with students, colleagues, and their respective families (Jennings and Greenberg, 2009). Thus, RE would be responsible for the effects. Third, the appraisal and expression of emotional abilities may overlap with the need for educational proficiency and subject matter understanding. For teachers to be more successful, they must become better at comprehending human emotion and displaying knowledge; those that possess such skills are more likely to have positive experiences in school (MacCann et al., 2014). In this case, the knowledge reserve of appraisal, understanding, and expression (a facet of EI) will have the most significant impact. We elaborate on the importance of these three mechanisms in the following sections.

### Mechanism 1: Is the regulation of emotion the key ingredient in emotional intelligence?

Teaching is an emotionally draining profession that can have negative effects on teachers’ well-being (Luque-Reca et al., 2022). In particular, rural teachers may be at risk due to the pressure of teaching and caring for students while learning new teaching skills. To obtain higher LS, such individuals must make the most of their positive emotions (Szczygieł and Mikolajczak, 2017). Negative emotions can have a similar effect, and those with higher RE can reduce the duration of such emotions, which can increase LS. Another possible explanation is that teachers who perceive themselves as being successful in managing their emotions may experience a sense of self-efficacy, which can lead to increased PA and higher LS (Vergara et al., 2015). Hence, the ability to regulate emotions is crucial for rural teachers. The model results from the EI facets suggest that PA and NA partially mediate the correlation between RE and LS.

### Mechanism 2: Does emotional intelligence affect life satisfaction through interpersonal processes?

There is evidence that higher EI is associated with stronger relationships within school settings (Lopes et al., 2004, 2005). Similarly, higher EI has been found to be an effective predictor of social support within the educational environment (Ciarrochi et al., 2001; Kong et al., 2012). Rural teachers with strong EI skills may be more capable of managing their lives and cultivating positive relationships with students, colleagues, and their respective families (Jennings and Greenberg, 2009). In the three facet-level subdimensional models, RE has the best model fit (AIC = 36407.088, BIC = 36707.779). As shown in the AEE and UE models, RE also has an indirect impact on LS through PA in addition to its direct impact, which is more likely to result from making it easier for rural teachers to access resources or improve achievement. Meanwhile, the indirect effect of RE on LS is more likely due to the help it provides to build a social support network for managing stress. According to the results, RE is more about improving PA, which has an indirect impact on LS. For example, teachers resonate with students in daily teaching to obtain more positive feedback, thereby improving their sense of accomplishment and LS.

### Mechanism 3: Is the appraisal and expression of emotion required for life satisfaction?

In Mechanism 3, we expected that the AEE facet of EI would be the strongest factor in predicting LS. However, the empirical model results did not support this assumption. In contrast, we found that the AEE model had the least predictive accuracy (AIC = 40826.774, BIC = 40162.328). Previous studies have shown that the perception and understanding facets of ability EI are the most important factors in predicting students’ academic performance in the school environment (MacCann et al., 2020). Therefore, we question why the AEE model has the least explanatory power for LS. We believe that the SREIT scale used in this study does not include the understanding-branch subscale, making the use of the scale different from previous research. The potential overlap between EI and teaching skills appears to be less important for their LS possibly because larger vocabularies and better expressive skills are not characteristic of teaching and school life in rural areas. Moreover, rural teachers are usually required to teach using textbooks and relying on examinations instead of exploratory teaching. Thus, the characteristics of this teaching mode do not necessitate more vocabulary knowledge and the ability to express oneself. In comparison, rural teachers may benefit more from having the ability to regulate and appropriately use emotions than from having a large vocabulary and expressive abilities.

## Limitations

This study has some limitations. First, the data were collected through self-reporting tools that may be somewhat subjective. Multiple assessment methods should be used to reduce this bias. Second, although our study used GLasso and SEM, two statistical methods with differing orientations, for data analysis and obtained a relatively robust correlation result, there may be some flaws in the causal inference of the conclusion due to the failure to use panel tracking data or standard randomized experimental data. Third, the results of this study can only be applied to rural teachers in central China, and there are insufficient cross-cultural inferences for the conclusions. In future research, we hope to use a more scientific research design and adopt superior statistical methods to conduct in-depth cross-cultural discussions on research issues. Fourth, because the participant data were collected through online questionnaires, there may be certain deviations due to the characteristics of Internet transmission and filling quality. However, we have considered various factors, such as school level, gender, and age, in the process of distributing the questionnaires; therefore, the participant data can reflect the situation of rural teachers in central China. Fifth, the participant data were collected in the fall semester of 2022, that is, during the COVID-19 pandemic, which may have caused teachers to face more pressure than ever before in teaching and life. In this regard, because the impact of EI on LS may vary, we look forward to conducting comparative studies between the pandemic and post-pandemic periods in future research. Finally, although we found that most of the teachers started teaching at the age of 22 years (after graduating with a bachelor’s degree), some of them began teaching at the age of 18 years. Considering the potential impact of teaching age on teachers’ LS and EI, the absence of this control variable could introduce some bias in the research results. Thus, future studies should focus on the significance of this variable.

## Implications

This study thoroughly explored how trait EI, PA, and NA relate to general LS. Instead of analyzing trait EI, PA, and NA as a single structure, this study focused on each trait EI substructure and the predictive ability of PA and NA and elucidated potential relationships among trait EI, PA, NA, and LS. By examining these specific facets of trait EI, PA, and NA, our results have implications for teacher groups in particular. Our findings may lead to the development of better individual and group staff counseling programs for teachers that take AEE, RE, UE, and their combined effects into account. Effective intervention strategies can be implemented to improve LS, and these programs can help teachers better cope with workplace stressors. Additionally, most research in this field focuses on Western culture. The results of this study have enriched the literature by providing additional empirical evidence regarding the relationship among trait EI, PA, NA, and LS, with a focus on Eastern cultures and rural school teacher groups. More specifically, rural school teachers in Eastern cultures tend to face more multifaceted job stress than teachers in Western cultures; they need to work with students from families with financial difficulties by accounting for the impact of different family backgrounds on student performance and by coping with other factors. Thus, the relationship between teachers’ emphasis on family and social factors and their personal LS was stronger. Future practitioners should keep this in mind when intervening with individuals from Eastern cultures.

## Data availability statement

The raw data supporting the conclusions of this article will be made available by the authors, without undue reservation.

## Author contributions

All authors listed have made a substantial, direct, and intellectual contribution to the work and approved it for publication.

## Funding

This study received funding from Fundamental Research Funds for the Central Universities (2022ECNU-HWCBFBLW004).

## Conflict of interest

The authors declare that the research was conducted in the absence of any commercial or financial relationships that could be construed as a potential conflict of interest.

## Publisher’s note

All claims expressed in this article are solely those of the authors and do not necessarily represent those of their affiliated organizations, or those of the publisher, the editors and the reviewers. Any product that may be evaluated in this article, or claim that may be made by its manufacturer, is not guaranteed or endorsed by the publisher.
